# The Late Ashbel Smith

**Published:** 1886-04

**Authors:** 


### THE LATE ASHBEL SMITH.

The admirable Moss engraving of this distinguished personage, which adorns this issue of the JOURNAL, will be a most welcome and acceptable *souvenir* to all Texas, and especially to the Texas physicians, in whose hearts he held first place.

The complete and extensive biographical sketch which accompanies it—the first and only authentic one published, was written by Col. Jas. D. Lynch, of Mississippi, the talented author of "The Bench and Bar of Texas," at the request of Judge Goldthwaite and Dr. D. F. Stuart, two of Dr. Smith's most intimate friends, whom we had asked to prepare it. It is a valuable document, and doubtless will be preserved in the archives of the State—that portion of it especially, relating to the annexation of Texas, being heretofore unwritten history.

We have issued 500 extra copies of this edition, for distribution amongst our patrons. Others desiring a copy will please enclose the price—25 cents. Begin your subscription now,

($2 00 a year in advance), and secure a copy of this issue for your posterity.

